# Catechol-O-Methyltransferase (COMT) Gene (Val158Met) and Brain-Derived Neurotropic Factor (BDNF) (Val66Met) Genes Polymorphism in Schizophrenia: A Case-Control Study 

**Published:** 2017-10

**Authors:** Ramin Saravani, Hamid Reza Galavi, Marzieh Lotfian Sargazi

**Affiliations:** 1 Cellular and Molecular Research Center, Zahedan University of Medical Sciences, Zahedan, Iran.; 2 Department of Clinical Biochemistry, School of Medicine, Zahedan University of Medical Sciences, Zahedan, Iran.

**Keywords:** *BDNF*, *COMT*, *Schizophrenia*, *Single Nucleotide Polymorphism*

## Abstract

**Objective: **Several studies have shown that some polymorphisms of genes encoding catechol-O-methyltransferase (COMT), the key enzyme in degrading dopamine, and norepinephrine and the human brain-derived neurotropic factor (BDNF), a nerve growth factor, are strong candidates for risk of schizophrenia (SCZ). In the present study, we aimed at examining the effects of COMT Val158Met (G>A) and BDNF Val66Met (G>A) polymorphisms on SCZ risk in a sample of Iranian population.

**Method:** This case- control study included 92 SCZ patients and 92 healthy controls (HCs). Genotyping of both variants (COMT Val158Met (G>A) and BDNF Val66Met (G>A)) were conducted using Amplification Refractory Mutation System-Polymerase Chain Reaction (ARMS-PCR).

**Results:** The findings revealed that the COMT Val158Met (G>A) polymorphism was not associated with the risk/protective of SCZ in all models (OR=0.630, 95%CI=0.299-1.326, P=0.224, GA vs. GG, OR=1.416, 95%CI=0.719-2.793, P=0.314, AA vs. GG, OR=1.00, 95%CI=0.56-1.79, P=1.00 GA+AA vs. GG, OR=1.667, 95%CI=0.885-3.125, P=0.11, AA vs. GG+GA, OR=1.247, 95%CI=0.825-1.885, P=0.343, A vs. G,). However, BDNF Val66Met (G>A) variant increased the risk of SCZ (OR = 2.008 95%CI = 1.008-4.00, P = 0.047, GA vs. GG, OR = 3.876 95%CI = 1.001-14.925, P = 0.049. AA vs. GG, OR = 2.272. 95%CI = 1.204-4.347, P = 0.011, GA+AA vs. GG, OR = 2.22 95%CI = 1.29-3.82. P = 0.005, A vs. G).

**Conclusion:** The results did not support an association between COMT Val158Met (G>A) variant and risk/protective of SCZ. Moreover, it was found that BDNF Val66Met (G>A) polymorphism may increase the risk of SCZ development. Further studies and different ethnicities are recommended to confirm the findings.

Schizophrenia (SCZ) is a complex psychiatric disorder that affects nearly 1% of the general population worldwide characterized by hallucinations, delusions, and incompatibility of mental activity and environment; however, the exact mechanisms underlying the development and progression of SCZ are still entirely unknown, but genetic factor accounts for more than 80% of etiology and heritability of SCZ (1-3).

The human Catechol-O-methyltransferase (COMT) gene is located on the long arm of Chromosome 22 at Position 11.21 (22q11) microdeletion, COMT with transferring a methyl group to catecholamine due to their degrading including dopamine, adrenaline, and noradrenaline (4, 5). 

cted on the association of COMT gene as a candidate gene for SCZ, so the activity of this enzyme is regulated by a common polymorphism causing substantial variations in the activity of this enzyme (4, 6, 7).

A functional single nucleotide polymorphism (SNP) with code rs4680G>A causes guanine change to adenine (exchange between nucleotide) in the gene sequence, which produces methionine amino acid from valine amino acid (Val>Met) at Codon 158. This change, which is due to the reduction of the activity of COMT enzyme with Met vs. Val (one-fourth of the enzymatic activity), results in a slower degradation of dopamine and greater availability (8). In addition, Chen et al. reported the homozygote “Met” Gas 3 to 4 fold lower enzymatic activity compared with the homozygote “Val”, while the heterozygote has approximately intermediate activity (9, 10). Moreover, a pervious study showed that Val allele of this SNP was associated with cognitive characterization of SCZ patients such as poor performance in tests of working memory (11). Moreover, this SNP was associated in some ethnics, while the same association did not exist in other populations (12, 13). The human brain-derived neurotropic factor (BDNF) gene, located on the short arm of chromosome 11 at Position 14.1 (11p14.1), is one member of the neurotropic factor family that promotes the development, survival, regeneration, and maintenance of the function of neurons. Several roles have been demonstrated for BDNF on the importance of BDNF such as modulate neurotransmitter, synthesis, metabolism, long-term potentiation, and postsynaptic ion channel fluxes (14). Several studies have found a change in the level of BDNF in hippocampus and the cortical area of SCZ patients; moreover, they reported that BDNF plays an important role in neurotransmission. Abundant genetic studies have shown the possible correlation between BDNF gene polymorphisms and SCZ even though some studies have not found any correlation between BDNF Val66Met polymorphism and psychiatric disorder. Furthermore, it has been demonstrated that Val66Met polymorphism of BDNF affects human memory and hippocampal function (14-17). Therefore, considering the possible importance of these 2 proteins in SCZ susceptibility and their relationship with SCZ, which have not been evaluated in any sample of Iranian population to date, in the present study we aimed at finding a relationship between COMT Val158Met (G>A) rs4680 and BDNF Val66Met (G>A) rs6265 polymorphisms and SCZ in a sample of Iranian population (Southeast of Iran).

## Materials and Methods

This case-control study was performed on 92 patients with SCZ who referred to Baharan hospital in the city of Zahedan, Iran from June 2015 to June 2016). Their disease was diagnosed by well-trained psychiatrists according to the Diagnostic and Statistical Manual of Mental Disorder, fourth edition (American, Psychiatric Association 1994) and previous works of the authors (18, 19). A total of 92 healthy blood donors without any history of systematic diseases, psychiatric disorders, asthma, cancer, cataracts, or psychotic disorder with major depression, schizophrenia, and bipolar disorder were selected. The case group was age and gender matched with the healthy controls (HCs) for a routine annual checkup from Ali-asghar hospital (Zahedan, Iran).

*Ethics*

The local ethics committee of Zahedan University of Medical Sciences (ethical code: 7716) approved the study protocol. Informed consent was obtained from all participants (and the next of kin) before participating in this study.

*Genomic DNA Extraction*

Genomic DNA was extracted from whole blood (2mL in EDTA-containing tubes) of the patient and control groups immediately after collection using salting-out method as described previously (20). The electrophoresis, NanoDrop, and a spectrophotometer (WPA, UK) (based on a ratio of 260/280) were used to examine the quality and quantity of the extracted DNA. The DNA samples were stored at -20ºC until analysis.

*PCR Reaction*

The analysis of COMT Val158Met (G>A) and BDNF Val66Met (G>A) polymorphisms were performed by Amplification Refractory Mutation System- Polymerase Chain Reaction (ARMS-PCR). The list of primers, designed by using Wasp primer software, (Pishgam Company, Tehran, Iran) used for amplification of SNPs is provided in Table 1. The final PCR mixture volume was 20μL consisting of 2 μL of genomic DNA (~80-100 ng), 1.5 μL of each primer (10-pmol/μL), 10μL master mix (Ampliqon Taq 2x mastermix, Denmark), and 5 μL of DNase-free water (SinaClon BioScience Co., Tehran, Iran). Eppendorf thermal cycler (Eppendorf AG, Hamburg, Germany) was used for amplification with the following condition: COMT Val158Met (G>A): an initial denaturation at 94 ℃ for 5 minutes followed by 30 cycles at 94 ℃ for 30 seconds; annealing 49℃ for 30 seconds; extension at 72 ℃ for 30 seconds and at the end of the 30th cycle; the final extension was at 72 ℃ for 5 minutes. The BDNF Val66Met (G>A) condition was similar to the above conditions except for the annealing temperature, which was 62 ℃.

*Gel Electrophoresis*

PCR products were verified on 1.5% of agarose (Invitrogen Life Technologies, Gaithersburg, MD, USA) containing ethidium bromide (0.7μg/ml) (Sigma-Aldrich, Steinheim, Germany) observed under UV light. The sizes of PCR products were reported to be 244bp for COMT Val158Met (G>A) and 258bp for BDNF Val66Met (G>A). At least, 20% of samples, either SCZ or HCs, were regenotyped randomly, and the new results confirmed the past results.

*Statistical Analysis*

Statistical analyses were performed using SPSS statistical software Version 22 (SPSS, Chicago, IL, USA). The rates of the genotypes and alleles were compared using the binary logistic regression and Mann-Whitney test. Categorical data were compared using Pearson’s *X*^2^ test. A probability of p<0.05 was considered statistically significant.

## Results

The demographic characteristics of the patients with SCZ and HCs are provided in Table 2. The mean age and gender were not significantly different between the 2 groups (P>0.05). There were no significant differences between the 2 groups in other parameters such as the level of education, place of residence, and number of family members (P>0.05).

The frequencies of alleles and genotype of COMT Val158Met (G>A) and BDNF Val66Met (G>A) polymorphisms are presented in Table 3.The frequencies of allele and genotypes of COMT Val158Met (G>A) polymorphism were not significantly different between the patients with SCZ and HCs (P>0.05). Moreover, no differences were observed between the 2 groups in dominant and recessive models of COMT Val158Met (G>A) (P>0.05). The results (Table 3) indicated that BDNF Val66Met (G>A) genotype significantly increased the risk of SCZ in the codominant model (P = 0.047, GA vs. GG and P = 0.049, AA vs. GG). Furthermore, an allele of this SNP was associated with 2.2 fold higher risk of SCZ (OR = 2.2, 95%CI = 1.29-3.82. P = 0.005). 

In addition, BDNF Val66Met (G>A) in the dominant (GA+AA vs. GG) model indicated an increased risk of SCZ (OR = 2.272. 95%CI = 1.204-4.347. P = 0.011).

 As demonstrated in Table 4, significant associations weren’t found between these two SNPs and risk of SCZ in both genders separately, except for allele frequency of BDNF polymorphism in the female group (A vs. G, p = 0.023).

The BDNF Val66Met (G>A) polymorphism conformed to the Hardy Weinberg Equilibrium (HWE) in the SCZ patients and HCs (*X*^2^ = 3.27, P = 0.07 and *X*^2^ = 1.74, P = 0.19, respectively), another SNP, COMT Val158Met (G>A), did not confirm HWE (case, *X*^2^ = 36.32, P<0.001 and HCs, *X*^2^ = 14.16, P<0.001).

Moreover, the possible association between both variants and clinical and demographic characteristics of SCZ patients in the dominant model was evaluated (Table 5); no significant association was observed between genotypes of either COMT Val158Met (G>A) or BDNF Val66Met (G>A) and clinical and demographic data (P>0.05), except for age, in BDNF Val66Met (G>A) (P = 0.002).

## Discussion

The findings revealed no association between COMT Val158Met (G>A) and SCZ, while the BDNF Val66Met (G>A) variant was associated with the risk of SCZ, either genotype or allele. SCZ is a multifactorial disease with unclear and unknown etiology, but several factors are possibly involved in the SCZ incidence, prevalence, and mortality such as sex, migration status, urban status, economic status, and latitude (21). It is suggested that genetic polymorphisms in COMT Val158Met (G>A) and BDNF Val66Met (G>A) may affect the pathogenesis of SCZ. In this study, the possible association between BDNF Val66Met (G>A) and COMT Val158Met (G>A), and the risk of SCZ were investigated in a sample of Iranian population (Southeast Iran). To the best of our knowledge, no study has examined the effect of the COMT Val158Met (G>A) and BDNF Val66Met (G>A) and the risk of SCZ on the Iranian population.

**Table 1:** Allele-Specific Polymerase (ASP), Polymerase Chain Reaction Primers sequences

**Primer 5’-3’**	**Product**	**Method**
COMT Val158Met (G/A) rs4680:	244bp	ARMS
RW:GCATGTACACCTTGTCCTTCTT		
RM:GCATGTACACCTTGTCCTTCTC		
F:CACCTGTGCTCACTTCTC		
BDNF Val66Met (G/A) rs6265	258bp	ARMS
FW:ATTGGCTGACACTTTCGAACAAA		
FM:ATTGGCTGACACTTTCGAACAAG		
R:ATACTGTCACACACGCTCAG		

COMT; Catechol-O-methyl Transferase, BDNF; the human brain-derived neurotropic factor, bp; Base Pair, ARMS; Amplification Refractory Mutation System, R; Reverse, F; Forward, W; Wild, M; Mutant

**Table 2:** Demographic characteristics of SCZ patients and controls

	SCZ (n=92)	HCs (n=92)	P-value
Age (year)	35.97±11.51	36.16±11.23	0.809
Sex (female/male)	38/54	38/54	1.000
Level of education (illiterate/literacy)	18/74	19/73	0.854
Place of residence (urban and rural)	59/33	57/35	0.760
No. family members (mean ± SD)	6.38±2.11	6.19±2.29	0.597

SCZ; Schizophrenia

HCs; Healthy controls

**Table 3:** Genotypic and allelic frequencies of BDNF (Val66Met) and COMT (Val158Met) polymorphisms in SCZ patients and control subjects

**Gene polymorphisms**	**SCZ n (%)**	**HCs n (%)**	**OR (95%CI)**	**P-value**
COMT (Val158Met) rs4680				
Codominant				
GG	41(44.6%)	41(44.6%)	Ref	
GA	17(18.4%)	27(29.4%)	0.630(0.299-1.326)	0.224
AA	34(37%)	24(26.1%)	1.416(0.719-2.793)	0.314
Allele				
G	99(54%)	109(59%)	Ref	
A	85(46%)	75(41%)	1.247(0.825-1.885)	0.343
Dominant				
GG	41(44.6%)	41(44.6%)	Ref	
GA+AA	51(55.4%)	51(55.4%)	1.00(0.56-1.79)	1.00
Recessive				
GG+GA	58(63%)	68(73.9%)	Ref	
AA	34(37%)	24(26.1%)	1.667(0.885-3.125)	0.11
BDNF (Val66Met) rs6265				
Codominant				
GG	55(59.8%)	71(77.2%)	Ref	
GA	28(30.4%)	18(19.6%)	2.008(1.008-4)	0.047
AA	9(9.8%)	3(3.3%)	3.876(1.001-14.925)	0.049
Allele				
G	138(75%)	160(87%)	Ref	
A	46(25%)	24(13%)	2.22(1.29-3.82)	0.005
Dominant				
GG	55(59.8%)	71(77.2%)	Ref	
GA+AA	37(40.2%)	21(22.8%)	2.272(1.204-4.347)	0.011
Recessive				
GG+GA	83(90.2%)	89(96.7%)	Ref	
AA	9(9.8%)	3(3.3%)	3.226(0.840-12.5)	0.067

SCZ; Schizophrenia, HCs; Healthy controls, COMT; Catechol-O-methyl Transferase, BDNF; the human brain-derived neurotropic factor, CI; = Confidence Intervals, OR; Odds Ratio

**Table 4:** Genotypic and allelic frequencies of BDNF (Val66Met) and COMT (Val158Met) polymorphisms in SCZ patients and control subjects in male and female as separately

**Gene polymorphisms**	**SCZ n (%)**	**HCs n (%)**	**OR (95%CI)**	**P-value**
COMT (Val158Met) rs4680				
Male				
GG	21(38.9)	26(48.1)	Ref.	-
GA	9(16.7)	13(24.1)	0.857(0.307-2.392)	0.768
AA	24(44.4)	15(27.8)	1.980(0.835-4.695)	0.121
G	51(47.2)	65(60.2)	Ref.	-
A	57(52.8)	43(39.8)	1.689(0.984-2.898)	0.0758
Female				
GG	20(52.6)	15(39.5)	Ref.	-
GA	8(21.1)	14(36.8)	0.428(0.143-1.284)	0.130
AA	10(26.3)	9(23.7)	1.200(0.391-3.686)	0.750
G	48(63.2)	44(57.9)	Ref.	-
A	28(36.8)	32(42.1)	0.618(0.418-1.538)	0.618
BDNF (Val66Met) rs6265				
Male				
GG	33(61.1)	41(75.9)	Ref.	-
GA	17(31.5)	11(20.4)	1.919(0.792-4.651)	0.149
AA	4(7.4)	2(3.7)	2.487(0.428-14.49)	0.310
G	83(76.9)	93(86.1)	Ref.	-
A	25(23.1)	15(13.9)	1.867(0.922-3.780)	0.114
Female				
GG	22(57.9)	30(78.9)	Ref.	-
GA	11(28.9)	7(18.4)	2.141(0.264-6.410)	0.173
AA	5(13.2)	1(2.7)	6.802(0.743-62.5)	0.090
G	55(72.4)	67(88.2)	Ref.	-
A	21(27.6)	9(11.8)	2.842(1.204-6.706)	0.023

SCZ; Schizophrenia, HCs; Healthy controls, COMT; Catechol-O-methyl Transferase, BDNF; the human brain-derived neurotropic factor, CI; = Confidence Intervals, OR; Odds Ratio

**Table 5:** Association between COMT and BDNF polymorphisms with clinical demographic and characteristics of SCZ patients

Genotype	Age(year)	Sex(Male/Female)	Hallucination(Yes/No)	Delirium(Yes/No)
COMT (Val158Met) rs4680				
GG	35.31±11.15	21/20	32/9	34/7
GA+AA	36.51±11.87	33/18	38/13	42/9
P-value	0.624	0.209	0.807	1.00
BDNF (Val66Met) rs6265				
GG	33.02±9.71	33/22	45/10	47/8
GA+AA	40.38±12.67	21/16	25/12	29/8
P-value	0.002	0.830	0.139	0.411

COMT; Catechol-O-methyl Transferase, BDNF; the human brain-derived neurotropic factor

COMT enzyme is an important degradation enzyme involved in the metabolism of catecholamine, consisting of dopamine, adrenaline, and noradrenaline. Evidence shows that polymorphism of COMT gene may influence the activity of COMT enzyme; consequently, the effectiveness of COMT enzyme decreases, and slower degradation of dopamine may be involved in SCZ development (10, 22). Another gene, BDNF, is a member of the neurotropic factor family and plays a key role in the development, regeneration of neurons, and metabolism and synthesis of neurotransmitters in the brain (16). Previous studies have shown an association between polymorphism and alternation of BDNF level in patents with SCZ (14, 23).

Similarly, several investigations have indicated an association between BDNF Val66Met (G>A) polymorphism and SCZ, confirming the results of the current study. Naoe et al. performed a meta-analysis on 8 case control studies including 2156 Caucasians with SCZ and 3007 HCs and suggested that Val66Met (G>A) polymorphism of BDNF gene was significantly associated with SCZ risk (16). Neves-Pereira et al. reported that BDNF Val66Met (G>A) variant was associated with increased risk of SCZ in Scots population, either genotype or allele (24). However, in

a case-control study performed by Chen et al. on Han Chinese people, BDNF Val66Met (G>A) polymorphism was not associated with SCZ development (15). Also, Watanabe et al. did not find any correlation between BDNF Val66Met (G>A) and SCZ in a sample of Japanese population (17). 

Contradictory to the current results, Tochigi et al. did not find any association between BDNF Val66Met 

 (G>A) variant and risk of SCZ susceptibility in Japanese population (25). Chen-jee Hong et al. concluded that the carriage of GA genotype of Val66Met (G>A) polymorphism of BDNF gen was associated with SCZ in Taiwanese population (15). Similarly, there was a relationship between BDNF 

Val66Met (G>A) and SCZ in several case-control and meta-analysis studies in Asia (14, 26, 27). BDNF Val66Met polymorphism is associated with aggressive behavior in schizophrenia. Also, an association was reported between BDNF Met (/A) allele polymorphism and increased aggressive behavior in SCZ patients (28).

Several studies have also shown no association between COMT Val158Met (G>A) polymorphism and SCZ, approving the findings of the present study. Daniels did not find any correlation between COMT Val158Met (G>A) and SCZ in Wales (29). Similarly, Chenet et al. (30), Karayiorgou et al. (31), and Daniels et al. (29) found no significant association between Val158Met of COMT gene polymorphism and risk of SCZ in Chinese, European, and United Kingdom (UK) populations, respectively. Also, in another investigation conducted on a sample of UK population, Norton et al. reported no significant differences in genotype or allele frequencies between SCZ and HCs for Val158Met of COMT gene polymorphism (5). However, there are contradictory results; for example, Yan Wang et al. found that SNP COMT Val158Met (G>A) was significantly associated with blunted effect in Chinese with Han origin (32). In a case-control study on North American Japanese, Ohmori et al., (1998) found a significant relationship between SCZ and COMT Val158Met (G>A) (33). Also, regarding the genotype and allele of SNP, Sarah Tosato et al. found homozygosity of COMT Met/Met patients of this variant with SCZ, who had higher aggressive behavior compared to homozygosity of Val/Val individuals (34). Ali Sazci found that the Val/Met heterozygosity of COMT Val158Met (G>A) genotype significantly decreased the risk of SCZ in a Turkish population (OR = 0.641 95%CI = 0.468-0.878. P = 0.006) compared to the Val/Val COMT Val158Met (G>A) genotype. In contrast, Met/Met genotype increased the risk of SCZ compared to the reference genotype (Met/Met vs. Val/Val, OR = 2.085, 95%CI = 1.35-3.229, P = 0.001) (35). The discrepancies between these investigations concerning both SNPs may be related to environmental and genetic differences between ethnical groups and populations. 

In our population, Hardy-Weinberg disequilibrium could be due to several reasons such as the structure of population in our area (different ethnic groups), migration, small sample size, and consanguineous marriages.

The current investigation had several limitations. First, based on the published and Medline data, several COMT and BDNF polymorphisms have been identified in humans, while we investigated only 1 polymorphism from each gene. Second, we did not consider the differences according to specific schizophrenia subtypes. Third the small sample size.

## Conclusion

In conclusion, our results revealed a significant association between BDNF Val66Met (G>A) polymorphism and SCZ risk, not supporting an association between COMT Val158Met (G>A) and risk of SCZ in the population of Southeast Iran. Further studies with larger sample sizes and different ethnicities should be conducted to determine their effects on SCZ risk.
